# Quantum enhancement of accuracy and precision in optical interferometry

**DOI:** 10.1038/lsa.2017.163

**Published:** 2018-03-23

**Authors:** Florian Kaiser, Panagiotis Vergyris, Djeylan Aktas, Charles Babin, Laurent Labonté, Sébastien Tanzilli

**Affiliations:** 1 Université Côte d’Azur, Institut de Physique de Nice (INPHYNI), Nice 06108, France; 2 Now at 3. Physikalisches Institut, Universität Stuttgart, Stuttgart 70569, Germany; 3 Now at Center for Integrated Quantum Science and Technology (IQST), Stuttgart 70569, Germany; 4 École Normale Supérieure de Lyon, Lyon 69364, France

**Keywords:** chromatic dispersion, interferometry, quantum optics, quantum metrology

## Abstract

White-light interferometry is one of today’s most precise tools for determining the properties of optical materials. Its achievable precision and accuracy are typically limited by systematic errors due to a high number of interdependent data-fitting parameters. Here, we introduce spectrally resolved quantum white-light interferometry as a novel tool for optical property measurements, notably, chromatic dispersion in optical fibres. By exploiting both spectral and photon-number correlations of energy-time entangled photon pairs, the number of fitting parameters is significantly reduced, which eliminates systematic errors and leads to an absolute determination of the material parameter. By comparing the quantum method to state-of-the-art approaches, we demonstrate the quantum advantage of 2.4 times better measurement precision, despite requiring 62 times fewer photons. The improved results are due to conceptual advantages enabled by quantum optics, which are likely to define new standards in experimental methods for characterising optical materials.

## Introduction

Quantum technologies have received substantial attention as a means to improve the resolution and precision of metrological tasks by reducing statistical errors due to quantum noise^1, 2, 3, 4, 5, 6, 7, 8^. Far less attention has been given to their ability to reduce systematic errors. However, statistical and systematic errors are of equal importance in any measurement, and the latter are typically more difficult to characterise. Notable examples of quantum-improved measurements are the combination of multiple fundamental electronic quantum effects for a more accurate definition of the ampere^9^ and quantum-correlated ‘twin photon beams’ in establishing absolute and universal optical power standards^10^. In this letter we demonstrate a new use of quantum optics to reduce systematic errors in the technologically prominent application of spectrally resolved white-light interferometry (WLI). WLI is used for precise measurements of chromatic dispersion, that is, the second derivative of the wavelength-dependent optical phase. Classical WLI, however, requires precise interferometer equalization^11, 12^ and is influenced by third-order dispersion^13, 14^. This leads to systematic errors that are difficult to account for.

We eliminate these drawbacks by inferring chromatic dispersion using energy–time entangled photon pairs and coincidence counting to measure spectral correlation functions. In addition, we exploit photon–number correlations to achieve a twofold resolution enhancement. Our results demonstrate that this new strategy outperforms the precision and accuracy of previous quantum^15, 16^ and state-of-the-art techniques^11, 12^. Moreover, because our approach is essentially alignment-free, it enables the use of the same interferometer in a user-friendly manner for analysing a wide variety of different optical materials in terms of type, optical properties, length, etc.

### Standard WLI

**Figure 1:**
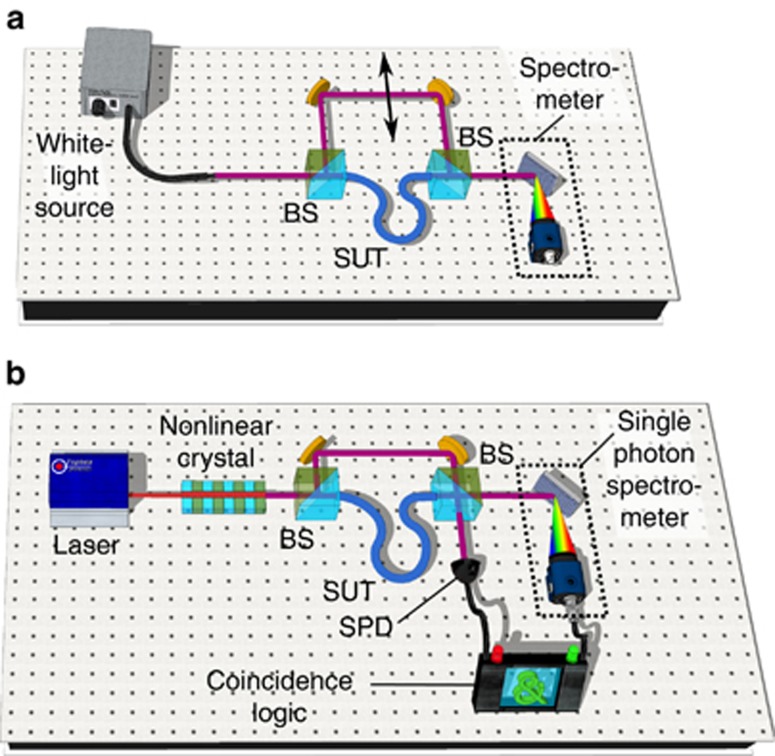
Typical experimental set-ups. (**a**) Standard spectrally resolved WLI. (**b**) Quantum WLI. BS, beam splitter.

The standard scheme for WLI is shown in Figure 1a. The emission of a white-light source is directed to an interferometer in which the reference arm is free-space (with well-known optical properties) and the other arm comprises the sample under test (SUT). Recombining both arms at the output beam splitter leads to an interference pattern for which the intensity follows *I*∝1+cos(*ϕ*(*λ*)), with 
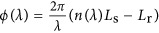
. Here, *λ* represents the wavelength, *L*_r_ and *L*_s_ are the physical lengths of the reference arm and the SUT, respectively, and *n*(*λ*) is the effective refractive index of the SUT. It is worth noting that interference is observed only when the interferometer is precisely balanced to within the larger of the coherence length of the white-light source and the coherence length imposed by the resolution of the spectrometer, which is typically on the order of microns to millimetres^11, 12^. In this case, the phase term reads (more details are given in the Supplementary Information):


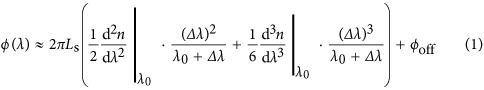


Here, *λ*_0_ represents the stationary phase point, that is, the wavelength at which the absolute phase difference between the two interferometer arms is exactly zero. In standard WLI, *λ*_0_ is extracted experimentally by identifying the symmetry point of the observed interferogram^11, 12^. Additionally, Δ*λ*=*λ*−*λ*_0_, and *ϕ*_off_ is a constant offset phase. Provided that *L*_s_ is precisely known, the optical material parameters 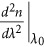 and 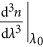 can be extracted from a fit to the data as a function of Δ*λ*. It is noteworthy that the three free parameters, that is, 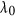, 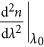 and 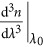, are usually all interdependent in a non-trivial manner such that uncertainties in one parameter systematically induce uncertainties in the others. In fact, the high number of fitting parameters required and the necessity to re-equilibrate the interferometer for every new SUT are the main limiting factors of this technique^13, 14^.

However, more accurate optical measurements are eagerly demanded in almost all fields involving optics. A special focus is made on the optical parameter 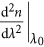, as it is directly related to the chromatic dispersion coefficient 
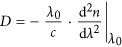
, where *c* is the speed of light^13, 17, 18, 19, 20, 21, 22, 23^. Chromatic dispersion causes optical pulse broadening, and more accurate measurements of *D* would have significant repercussions for optimising today’s telecommunication networks, developing new-generation pulsed lasers and amplifiers, and designing novel linear and nonlinear optical components and circuits, as well as for assessing the properties of biological tissues.

## Materials and methods

### Quantum WLI

Figure 1b depicts the new experimental schematic dedicated to spectrally resolved quantum WLI (Q-WLI) intended to overcome the above issues. The quantum white-light source is composed of a continuous-wave pump laser and a nonlinear crystal in which energy–time entangled photon pairs are generated through spontaneous parametric downconversion^24, 25^. This process obeys the conservation of the energy, that is, 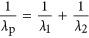. Here, *λ*_p,1,2_ respectively represent the wavelengths in vacuum of the pump laser photons and the individual photons for each generated pair. Another implication of the conservation of the energy is that the degenerate vacuum wavelength of the emission spectrum is λ*=2*λ*_p_. We send the paired photons to the interferometer; however, as opposed to standard WLI, we now intentionally unbalance it. This provides us with two advantages: first, we avoid single-photon interference, and second, we obtain a means to distinguish events in which the two photons take opposite paths (strongly delayed arrival times at the interferometer’s outputs) or the same path (near-zero arrival time difference)^24^. We postselect the latter events by considering only two-photon coincidence detection events in which both the single-photon detector (SPD) and the single-photon-sensitive spectrometer fire simultaneously. Our goal is now to observe quantum interference between these two-photon contributions, which necessitates that they be coherent and indistinguishable. Coherence is ensured by operating the interferometer at a path-length difference that is shorter than the coherence length of the pump laser (~100 m) such that the photon pair contributions are in phase^26^. Indistinguishability concerns mainly the temporal envelope of the photon pair wave packet, which is distorted from its original shape by the dispersion-induced temporal walk-off between the individual photons in the SUT. For standard fibres, this means that path-length differences up to ~10 m are acceptable^27^.

Thus, provided that the interferometer is operated in these conditions, near-zero arrival time coincidence detection results in a two-photon *N*00*N* state:


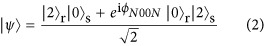


Here, the ket vectors, indexed by s and r, indicate the number of photons in the reference and SUT arms, respectively, and *ϕ*_*N*00*N*_=*ϕ*(*λ*_1_)+*ϕ*(*λ*_2_). We obtain the spectral dependence of *ϕ*_*N*00*N*_ by computing *ϕ*(*λ*_1_) and *ϕ*(*λ*_2_) according to Equation (1) and respecting the conservation of the energy during the downconversion process:


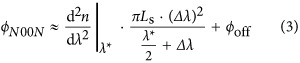


Here, 
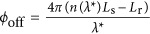
 is an offset term, and Δ*λ*=*λ*−λ*. The phase-dependent two-photon coincidence rate *R* is then *R*∝1+cos(*ϕ*_*N*00*N*_). In the past, numerous studies have investigated the term *ϕ*_off_, as it allows measuring optical phase shifts at constant wavelengths with double resolution compared to the standard approach^28, 29, 30^.

We access here, for the first time, the wavelength-dependent term in Equation (3) by recording *R* as a function of Δ*λ*; that is, the two-photon coincidence rate is measured as a function of the paired-photons’ wavelengths.

This leads to several pertinent purely quantum-enabled features. Due to the use of an energy–time entangled two-photon *N*00*N* state, the required precision of equilibrating the interferometer is ~10 m instead of microns to millimetres in standard WLI^11, 12, 13, 14^. This is particularly interesting for improving the ease of use, as no realignment is necessary when changing the SUT; compared to Equation (1), the third-order term 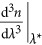 in Equation (3) is cancelled owing to energy–time correlations^16^. Furthermore, the wavelength at which chromatic dispersion is measured, λ*, need not be extracted from the data, as it is exactly twice the wavelength of the continuous-wave pump laser, *λ*_p_, and can therefore be known with extremely high accuracy. This means that the quantum strategy allows data fitting using exactly one free parameter, namely, 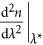, which is an essential step towards absolute optical-property determination with high precision without systematic errors. Finally, due to the use of a two-photon *N*00*N* state, double resolution of 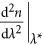 is achieved, enabling measurements on shorter samples and components compared to standard WLI, that is, down to the technologically interesting mm to cm scale.

### Detailed optical setup and data acquisition

To benchmark standard and quantum approaches, we used a 1-m long SMF28e fibre from Corning as the SUT. We used the same interferometer for all measurements and actively stabilised it using a reference laser and a piezoelectric transducer on one mirror in the reference arm (additional details are provided in the methods section). This ensured that *ϕ*_off_ remained constant.

For chromatic dispersion measurements using classical WLI, we used a state-of-the-art superluminescent diode. At the output of the interferometer we measured an average spectral intensity of ~125 pW nm^−1^ from 1450 to 1650 nm. Interferograms were recorded using a spectrometer from Anritsu (model MS9710B, Atsugi-shi, Japan) with 0.1 s integration time and 0.5 nm resolution, which are standard parameters for this kind of measurement^11, 12^.

For the Q-WLI approach, the light source was a 780.246 nm laser pumping a type-0 periodically poled lithium niobate waveguide. We stabilised the laser wavelength against the 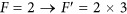 hyperfine crossover transition in atomic ^87^Rb such that *λ*_p_ and λ* were known with a precision of the order of 1 fm. The quasi-phase matching in the periodically poled lithium niobate waveguide was engineered to generate energy–time entangled photon pairs around the degenerate wavelength of λ*=1560.493 nm with a bandwidth of ∼140 nm^25^. To detect the paired photons, we used an InGaAs SPD (IDQ 220) at one interferometer output. The single-photon spectrometer at the other output was made of a wavelength-tunable 0.5 nm bandpass filter followed by another InGaAs SPD (IDQ 230). To avoid saturation of these detectors, the spectral intensity at the interferometer output was reduced to ∼25 fW nm^−1^, which was partially compensated by increasing the integration time to 8 s.

All measurements were repeated 100 times on the same SUT to infer the statistical accuracy of both WLI and Q-WLI approaches.

## Results and discussion

### Statistical analysis for comparing measurement precision

**Figure 2:**
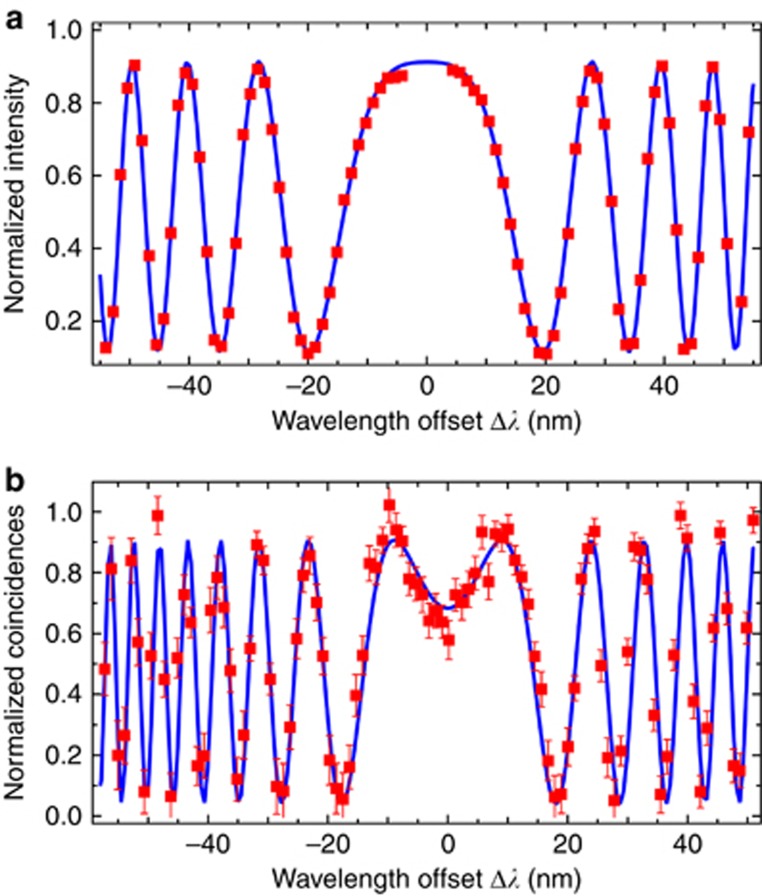
Typical measurements acquired for inferring chromatic dispersion in a 1-m-long standard single-mode fibre. (**a**) Results obtained with standard WLI, and (**b**) using Q-WLI. Red dots are data points; blue curves are appropriate fits to the data following Equations (1) and (3), from which *D* is extracted. Error bars assume Poissonian photon number statistics. For standard WLI, normalization was obtained by measuring two reference spectra. For Q-WLI, normalization was performed on the fly by counting non-zero arrival time difference coincidences. For more details, refer to the Supplementary Information.

**Figure 3:**
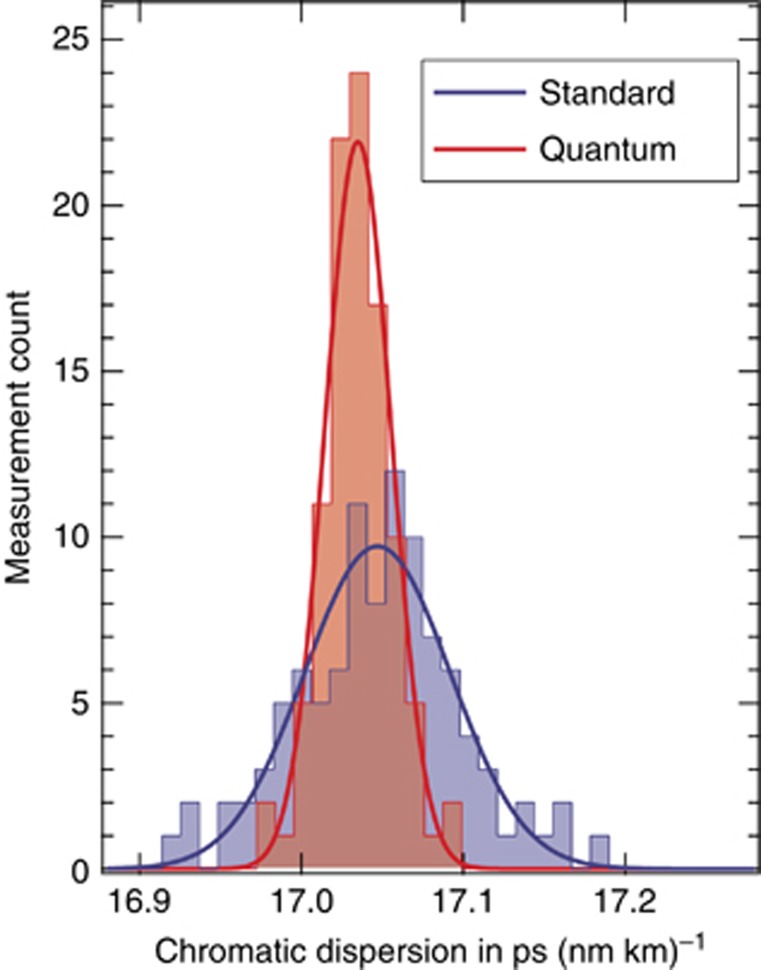
Histogram of inferred chromatic dispersion coefficients after 100 repetitions with the same SUT for both standard (blue) and quantum-enhanced (red) measurements. Fits to the data assumed a normal distribution.

Typical interference patterns for chromatic dispersion measurements using both methods are shown in Figure 2a and 2b. With the Q-WLI setup, we found twice as many interference fringes for the same spectral bandwidth, which is a direct consequence of the doubled phase sensitivity of the two-photon *N*00*N* state. After acquiring 2 × 100 measurements on the same SUT, we inferred the precisions of both approaches. The results of the statistical data analysis are shown in Figure 3. For standard WLI, we obtained, on average, 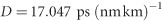 at *λ*_0_≈1560.5 nm with a standard deviation of 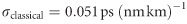. This result is among the most precise reported to date in the literature^13, 17, 18, 19, 20, 21, 22^. For Q-WLI, we measured, on average, 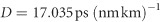 at λ*=1560.493 nm with a significantly smaller standard deviation of 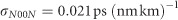.

In our two sets of data, we observed a difference of 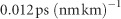 between the central values, which is larger than the deviation expected from statistical uncertainties 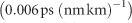. Polarization mode dispersion can be excluded as it would introduce at most an offset of 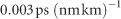. Consequently, the difference in central values must originate from systematic errors. In this sense, we compute that, for standard WLI, the difference can be explained by either a slight wavelength offset of the spectrometer (<0.2 nm) or by a slightly unbalanced interferometer (~1.5 μm). Both types of errors induce an error of the fitting parameter *λ*_0_ that translates to an error in 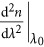 (Refs. 11, 12). At this point, we emphasise again that in our Q-WLI approach, λ* is known with essentially absolute accuracy, and an unbalanced interferometer does not influence the measurement. Because Q-WLI presents fewer sources of systematic errors, it is therefore natural to conclude that Q-WLI determines chromatic dispersion with absolute accuracy.

We further emphasise that our measurements performed with Q-WLI involve ~62 times fewer photons transmitted through the SUT compared to standard WLI. It is therefore interesting to compare the achievable precision normalised to the number of transmitted photons. For each standard and quantum interferogram, the number of photons reaching the interferometer outputs was *N*_std_≈2.0 × 10^10^ and *N*_quant_≈3.1 × 10^8^, respectively. Consequently, the standard and quantum methods achieve precisions of 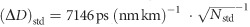 and 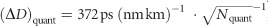, respectively. In other words, in addition to being more prone to systematic errors, the standard measurement requires 369 times more photons to achieve the same precision as Q-WLI.

### Device calibration using Q-WLI

Another advantage provided by Q-WLI lies in its straightforward device calibration. All of the optical components in the interferometer actually show small residual chromatic dispersion, and this undesired offset needs to be evaluated and subtracted from the data to avoid systematic errors. In both cases, this implies performing a measurement without any SUT.

Note that in standard WLI, removing the SUT significantly unbalances the interferometer, and to observe interference, the length of the reference arm must be reduced accordingly (typically on the order of 1 m). This procedure is technically challenging, time-consuming, and might lead to additional systematic errors.

**Figure 4:**
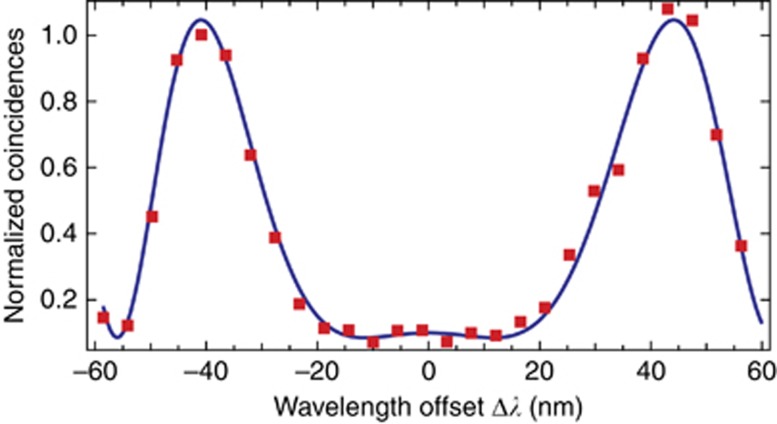
Experimental results when using Q-WLI for inferring residual chromatic dispersion in our interferometer without the SUT. Red dots, data points; the blue curve is an appropriate fit to the data following Equation (3).

At this point, Q-WLI demonstrates its ability for user-friendly operation. Even after removing the SUT, interference is observed without any interferometer realignment. Figure 4 shows the experimental results that we have obtained when measuring chromatic dispersion in our bare interferometer, that is, without the SUT. It turned out that in our interferometer, residual chromatic dispersion amounted to ~10% of the measured values on the 1 m SUT. For all of the data discussed above, except for the raw data in Figure 2a and 2b, we have subtracted the residual chromatic dispersion.

## Conclusions

We have introduced and demonstrated the concept of spectrally resolved Q-WLI, exploiting energy–time entangled two-photon *N*00*N* states. Compared to standard measurements, the *N*00*N* state permits achieving a phase sensitivity higher by a factor of two. More strikingly, this use of such quantum states of light reduces the number of free parameters for fitting experimental data from three to one, representing a major advantage for determining optical properties with high precision and absolute accuracy. In addition, our setup does not require a balanced interferometer for performing the measurement, which represents a significant time-saving advantage compared to standard WLI. This is of particular interest for device calibration and when measuring a large set of samples.

As an exemplary demonstration, we have applied our method to infer chromatic dispersion in a standard single-mode fibre, obtaining 2.4 times more precise results compared to state-of-the-art realizations, despite using ~62 times fewer photons.

We note that the sensitivity of our approach could be further doubled by using a double-pass configuration^18^; this could achieve measurements on short samples, such as optical components and waveguide structures (mm to cm length scale). Such measurements would also be of interest for medical applications where precise knowledge of chromatic dispersion in tissues is required to yield optimal image quality in optical coherence tomography^31^. From this perspective, the reduced number of photons required for quantum WLI is also highly interesting for measurements performed on photosensitive biological samples^32, 33, 34^. In optical telecommunication systems, by rotating the polarizations of the entangled photon pairs, our setup could be used for measuring polarization mode dispersion in optical components, which would lead to refinement of manufacturing processes.

In addition, total measurement times could be reduced far below 1 s by using high-speed superconducting detectors with ~3 orders of magnitude higher saturation levels compared to the InGaAs SPDs used here^35^. Alternatively, quantum-inspired strategies may also prove to be suitable^36, 37^.

In summary, we believe that combining the fundamental and conceptual advantages enabled by quantum light is a very promising approach for the future development and improvement of applications requiring absolute and high-precision measurements of optical properties.
