# Supplementary material for: Quantum enhancement of accuracy and precision in optical interferometry
Source: Light Sci Appl. 2018 Mar 23;7:17163–. doi: 10.1038/lsa.2017.163 (PMC6060044; doi:10.1038/lsa.2017.163)
Supplement: Supplementary Material [file lsa2017163x1.docx]

**Supplementary information for
Quantum enhancement of accuracy and precision in optical interferometry**

Florian Kaiser^1,2,3,*^, Panagiotis Vergyris^1^, Djeylan Aktas^1^, Charles Babin^1,4^, Laurent Labonté^1^, and Sébastien Tanzilli^1^

^1^ Université Côte d’Azur, Institut de Physique de Nice (INPHYNI), CNRS UMR 7010, Parc Valrose, 06108 Nice Cedex 2, France

^2^ Now at 3. Physikalisches Institut, Universität Stuttgart, Pfaffenwaldring 57, 70569 Stuttgart, Germany

^3^ Now at Center for Integrated Quantum Science and Technology (IQST), Stuttgart, Germany

^4^ École Normale Supérieure de Lyon, 46 Allée d’Italie, 69364 Lyon Cedex 07, France

^*^ Email: f.kaiser@physik.uni-stuttgart.de

**MACH-ZEHNDER INTERFEROMETER STABILISATION**

Without active interferometer phase stabilisation, we observe $2\pi$ phase drifts every few seconds due temperature drifts in the laboratory. This limits severely the integration times for both the classical and quantum measurements. Therefore, we employ an active phase stabilisation system. It is made of an actively wavelength-stabilised 1560.5 nm reference laser sent in the counter-propagating way through the interferometer, and a piezoelectric translation stage in the reference arm of the interferometer [SR1]. The feedback loop has a bandwidth of 100 Hz which results in a long-term phase stability of $<\frac{2\pi}{40} \mathrm{rad}$.

**SPATIAL AND POLARISATION MODE OVERLAP**

In order to obtain high-visibility interference patterns at the interferometer output, the photon (or photon pair) contributions from both interferometer arms need to be made indistinguishable in both the spatial and polarization modes. Spatial mode overlap is ensured by using a fibre-optic beam-splitter at the interferometer output and input [SR2]. Polarisation mode overlap is obtained using fibre-optic polarization controllers in both interferometer arms. These components are not shown in the main text figures in order to simplify the reading of the manuscript.

**QUALITY OF THE ENTANGLED PHOTON PAIR SOURCE**

We infer the entanglement quality of our photon pair source in the following configuration. We fix the analysis wavelength of the spectrometer at 1550 nm and postselect the desired $N00N$-state by a coincidence measurement. Then, the path length difference of the MZI is scanned and the two-photon coincidence rate is recorded. We measure sinusoidal oscillations with a raw fringe visibility of $87.1\pm2.2\%$ which increases to $95.5\pm2.6\%$ after the subtraction of detectors’ dark counts. In other words, we obtain a fidelity of 97.8% to the desired $N00N$-state. We explain imperfections by unbalanced losses between the two arms of the interferometer and multi-pair contributions.

**NORMALISATION OF INTENSITY AND COINCIDENCE SPECTROGRAMS**

For the classical strategy, normalisation is obtained by recording two reference spectrograms with either interferometer arm being blocked. Normalisation is obtained by dividing the data by the sum of both reference spectrograms.

For the entanglement-enabled strategy, we normalise the coincidence counts by taking advantage of the undesired contributions in which the paired photons take opposite paths inside the interferometer. These contributions do not interfere at the interferometer output, such that the related (non-zero time delay) coincidence rate is directly proportional to the spectral intensity of the photon pair generator. Normalisation is obtained by dividing the $N00N$-state coincidences by two times the sum of the non-$N00N$-state coincidences.

**HOME-MADE SINGLE-PHOTON SPECTROMETER**

As a single-photon spectrometer, we use a wavelength-tunable motorised bandpass filter (Yenista XTM-50) followed by a low noise single-photon avalanche photodiode (IDQ id230) operated at 25% detection efficiency. The transmission loss of the filter is measured to be 4 dB such that the total quantum efficiency of the single-photon spectrometer is $\sim10\%$.

**DERIVATION OF THE FITTING FUNCTION FOR STANDARD WLI**

As outlined in the manuscript, the wavelength dependent phase shift at the interferometer output is

$\phi\left( \lambda\right)=\frac{2\pi}{\lambda}\left( n\left( \lambda\right)\cdot L_{s}-L_{r} \right).$ ( S1 )

We approximate now $n(\lambda)$ by a third order Taylor series: $n\left( \lambda\right)=n\left( \lambda_{0}+\Delta\lambda\right)\approx n\left( \lambda_{0} \right)+\sum_{k=1}^{3} \frac{1}{k!}\left. \frac{d^{k}n}{{d\lambda}^{k}} \right|_{\lambda_{0}}\cdot{(\Delta\lambda)}^{k}$.

This leads to

$\phi\left( \lambda_{0}+\Delta\lambda\right)\approx2\pi L_{s}\left( \frac{n\left( \lambda_{0} \right)}{\lambda_{0}+\Delta\lambda}+\left. \frac{dn}{d\lambda} \right|_{\lambda_{0}}\cdot\frac{\Delta\lambda}{\lambda_{0}+\Delta\lambda}+\frac{1}{2}\left. \frac{d^{2}n}{{d\lambda}^{2}} \right|_{\lambda_{0}}\cdot\frac{\left( \Delta\lambda\right)^{2}}{\lambda_{0}+\Delta\lambda}+\frac{1}{6}\left. \frac{d^{3}n}{{d\lambda}^{3}} \right|_{\lambda_{0}}\cdot\frac{\left( \Delta\lambda\right)^{3}}{\lambda_{0}+\Delta\lambda} \right)-\frac{2\pi L_{r}}{\lambda_{0}+\Delta\lambda}.$ ( S2 )

Note that, in general, the interference fringes obtained at the interferometer output are usually too closely spaced to be resolved by a commercial spectrometer because of the strong phase-dependence on zero and first order derivatives. In order to cancel these terms, the interferometer has to be precisely equilibrated to the so-called stationary phase point (SPP), which is found at $L_{r}=\left( n\left( \lambda_{0} \right)-\left. \frac{dn}{d\lambda} \right|_{\lambda_{0}}\cdot\lambda_{0} \right)L_{s}$. Note that this point has to be found individually for each new sample with an accuracy on the order of a few micron. After finding the SPP, the dominant term is $\left. \frac{d^{2}n}{{d\lambda}^{2}} \right|_{\lambda_{0}}$ and the phase term simplifies to

$\phi\left( \lambda_{0}+\Delta\lambda\right)\approx2\pi L_{s}\left( \frac{1}{2}\left. \frac{d^{2}n}{{d\lambda}^{2}} \right|_{\lambda_{0}}\cdot\frac{\left( \Delta\lambda\right)^{2}}{\lambda_{0}+\Delta\lambda}+\frac{1}{6}\left. \frac{d^{3}n}{{d\lambda}^{3}} \right|_{\lambda_{0}}\cdot\frac{\left( \Delta\lambda\right)^{3}}{\lambda_{0}+\Delta\lambda} \right)+\phi_{off},$ ( S3 )

in which $\phi_{\mathrm{off}}=2\pi L_{s}\left. \frac{dn}{d\lambda} \right|_{\lambda_{0}}$ is a constant phase offset. Assuming that $L_{s}$ is known precisely, the required fitting parameters are therefore $\left. \frac{d^{2}n}{{d\lambda}^{2}} \right|_{\lambda_{0}}$, $\left. \frac{d^{3}n}{{d\lambda}^{3}} \right|_{\lambda_{0}}$ and $\lambda_{0}$.

**DATA FITTING UP TO** $\left. \frac{\boldsymbol{d}^{\boldsymbol{2}}\boldsymbol{n}}{\boldsymbol{d\lambda}^{\boldsymbol{2}}} \right|_{\boldsymbol{\lambda}_{\boldsymbol{0}}}$**and** $\left. \frac{\boldsymbol{d}^{\boldsymbol{3}}\boldsymbol{n}}{\boldsymbol{d\lambda}^{\boldsymbol{3}}} \right|_{\boldsymbol{\lambda}_{\boldsymbol{0}}}$

Data obtained with standard WLI require a fitting function taking into account terms up to $\left. \frac{d^{3}n}{{d\lambda}^{3}} \right|_{\lambda_{0}}$ to obtain the most precise and accurate results.

Fitting the data with the function described in equation S3 leads to $D=17.047 \frac{\mathrm{ps}}{nm\cdot km}$ at $\lambda_{0}\approx1560.5 \mathrm{nm}$ and $\sigma_{\mathrm{classical}}=0.051 \frac{\mathrm{ps}}{nm\cdot km}$ after 100 measurements on the same standard single-mode fibre.

A fitting function taking into account only terms up to $\left. \frac{d^{2}n}{{d\lambda}^{2}} \right|_{\lambda_{0}}$ does not lead to a good overlap between data and experiment (see Figure S1) which leads to both an offset and a larger standard deviation, i.e. $D=17.070 \frac{\mathrm{ps}}{nm\cdot km}$ and $\sigma_{\mathrm{classical}}=0.054 \frac{\mathrm{ps}}{nm\cdot km}$.

Figure S1. Fitting standard WLI data (red dots) with a function taking into account terms up to $\left. \frac{d^{2}n}{{d\lambda}^{2}} \right|_{\lambda^{*}}$ (green line) and $\left. \frac{d^{3}n}{{d\lambda}^{3}} \right|_{\lambda^{*}}$ (blue line). Only the latter shows a perfect overlap with the experimental data.

**DERIVATION OF THE FITTING FUNCTION FOR Q-WLI**

For the Q-WLI, the phase term is given by the two-photon phase $\phi_{N00N}=\phi\left( \lambda_{1} \right)+\phi\left( \lambda_{2} \right)$, which can be calculated using equation S2. Respecting the conservation of the energy, i.e., $\frac{1}{\lambda_{p}}=\frac{2}{\lambda^{*}}=\frac{1}{\lambda_{1}}+\frac{1}{\lambda_{2}}$, and setting $\lambda_{2}=\lambda^{*}+\Delta\lambda$ leads to

$\phi_{N00N}\left( \lambda^{*}+\Delta\lambda\right)\approx2\pi L_{s}\left( \frac{1}{2} \left. \frac{d^{2}n}{{d\lambda}^{2}} \right|_{\lambda^{*}}\cdot\frac{\left( \Delta\lambda\right)^{2}}{\frac{\lambda^{*}}{2}+\Delta\lambda}+\frac{1}{6} \left. \frac{d^{3}n}{{d\lambda}^{3}} \right|_{\lambda^{*}}\cdot\frac{{(\Delta\lambda)}^{4}}{\left( \frac{\lambda^{*}}{2}+\Delta\lambda\right)^{2}} \right)$ ( S4 )

where we consider the phase offset $\phi_{\mathrm{off}}=\frac{4\pi(n\left( \lambda^{*} \right)L_{s}-L_{r})}{\lambda^{*}}$ to be constant due to the active phase stabilisation system. Note that, compared to the classical phase term, the dependence on $\left. \frac{d^{3}n}{{d\lambda}^{3}} \right|_{\lambda^{*}}$ is strongly suppressed for $\Delta\lambda<\lambda^{*}$ which is generally the case. Therefore, $\left. \frac{d^{3}n}{{d\lambda}^{3}} \right|_{\lambda^{*}}$ does not have to be considered as a fitting parameter in Q-WLI.

**SUPPLEMENTARY REFERENCES**

| [SR1] | F. Kaiser, L. A. Ngah, A. Issautier, T. Delord, D. Aktas, *et al*, Polarization entangled photon-pair source based on quantum nonlinear photonics and interferometry, *Opt Commun* 2014; **327**: 7-16. |
| --- | --- |
| [SR2] | J. Y. Lee and D. Y. Kim, Versatile chromatic dispersion measurement of a single mode fiber using spectral white light interferometry, *Opt Express* 2006; **14**: 11608-11615. |
